# Impacts of language barriers on healthcare access and quality among Afaan Oromoo-speaking patients in Addis Ababa, Ethiopia

**DOI:** 10.1186/s12913-023-09036-z

**Published:** 2023-01-16

**Authors:** Amanti Baru Olani, Ararso Baru Olani, Takele Birhanu Muleta, Dame Habtamu Rikitu, Kusa Gemeda Disassa

**Affiliations:** 1 Department of Sociology, Jimma University, Jimma, Ethiopia; 2 College of Medicine and Health Sciences, Arbaminch University, Arbaminch, Ethiopia; 3 Slum and Rural Health Initiative Ethiopia, Addis Ababa, Ethiopia; 4 All Africa TB and Leprosy Research and Training (ALERT) Center Comprehensive Specialized Hospital, Addis Ababa, Ethiopia; 5 Department of Oromo Folklore and Literature, Jimma University, Jimma, Ethiopia

**Keywords:** Language barrier, Diversity, Healthcare access, Healthcare quality, Health inequality, Health and multinational federalism

## Abstract

**Background:**

Ethiopia is a multilingual and multinational federation with Addis Ababa serving as both the capital city of Oromia regional state and the seat of the Ethiopian federal government. Nevertheless, only Amharic is considered as the working language of the city and federal offices, including hospitals. As a result, Afaan Oromoo-speaking patients may be facing language barriers in the healthcare settings in Addis Ababa. Language barriers have the capacity to affect patients’ experience of care and treatment outcomes. This study, hence, examined the impacts of language barriers on the healthcare access and quality for the Afaan Oromoo-speaking patients in public hospitals in Addis Ababa.

**Methods:**

In-depth interviews with patients (*N* = 27) and key informant interviews with healthcare providers (*N* = 9) were conducted in six public hospitals found in Addis Ababa. All the interviews were audio-taped and transcribed verbatim. A thematic analysis technique was employed to address the study objectives.

**Results:**

The study participants indicated the widely existing problem of language discordance between patients and healthcare providers. The impacts of language barriers on the patients include preventable medical errors, low treatment adherence, low health-seeking behavior, additional treatment cost, increased length of hospital stays, weak therapeutic relation, social desirability bias, less confidence, and dissatisfaction with the healthcare. For the healthcare providers, language barriers are affecting their ability to take patient history, perform diagnoses and provide treatment, and have also increased their work burden. The use of ad hoc interpreters sourced from bilingual/multilingual patients, patient attendants, volunteer healthcare providers, and other casual people has been reported to deal with the problem of language barriers.

**Conclusion:**

A significant number of Afaan Oromoo-speaking patients are facing language barriers in accessing quality healthcare in public hospitals in Addis Ababa, and this constitutes structural violence. As a way out, making Afaan Oromoo an additional working language of the public hospitals in Addis Ababa, the assignment of professional interpreters, and a hiring system that promotes the recruitment of qualified multi-lingual healthcare providers are suggested.

**Supplementary Information:**

The online version contains supplementary material available at 10.1186/s12913-023-09036-z.

## Introduction

Good level of communication between the healthcare providers and patients is critical to ensure the quality of healthcare, the safety of patients and the satisfaction of both the patients and healthcare providers [1–3]. As a result, the communication between healthcare providers and the patient has long been recognized to be of diagnostic importance and treatment benefits [4]. In fact, every phase of the healthcare process, all the way from identification of the patient’s health problem and treatment to consultation and eventually termination of treatment is entirely dependent on effective communication [5]. “Just listen to your patient, he is telling you the diagnosis” is a popular saying in medicine to emphasize the cruciality of history-taking in the diagnostic process [6, 7] and Pitkin [7] further said “And she [the patient] just might be telling you the best management, too”.

Evidence shows that the healthcare providers’ ability to explain, listen and empathize with the patient’s feelings may has a significant effect on the disease outcomes and experience of care [4, 8, 9]. However, communication between the patient and healthcare providers can be impaired by language barriers [3, 10, 11]. Previous studies show that language barriers have negative consequences for linguistic minorities in both accessibility and quality of healthcare [2, 10, 12, 13]. Therefore, appropriate language services have the potential to address health disparities in accessing healthcare, and quality of care received such as understanding of medication instructions, procedures, treatment, and appointment visits [4, 11]. As a result, health systems are increasingly recognizing the need to promote cultural understanding and therapeutic alliance between healthcare providers and the patients, and advocating for the use of professional and regulated language access services to achieve the goal of overcoming the potential adverse events likely to occur from language barriers [14, 15]. ﻿Language access services include provision of service by bilingual providers, certified interpreters, signage, translated health information and digital technologies [15, 16].

Ethiopia has signed and ratified several international and regional human right conventions regarding the access to healthcare services. Of which, the World Health Organization (WHO) constitution says that every human being has the right to enjoyment of the highest attainable standard of health without distinction of race, religion, political belief, economic or social condition [17]. Similarly, according to article 16 of African charter on human and peoples’ rights, every individual shall have the right to enjoy the best attainable state of physical and mental health [18]. However, language barriers are limiting the accessibility of healthcare services and quality of care in Ethiopia [19, 20]. Ethiopia is a multilingual state practicing a multinational federation system since 1995. The federation is currently made up of 11 regional states and is a home to about 80 ethnonational groups having their own languages, of which the top five major ones are Oromo (35%), Amhara (27%), Somali (6.2%), Tigrayan (6.1%), and Sidama (4.0%) [21].

**Fig. 1:**
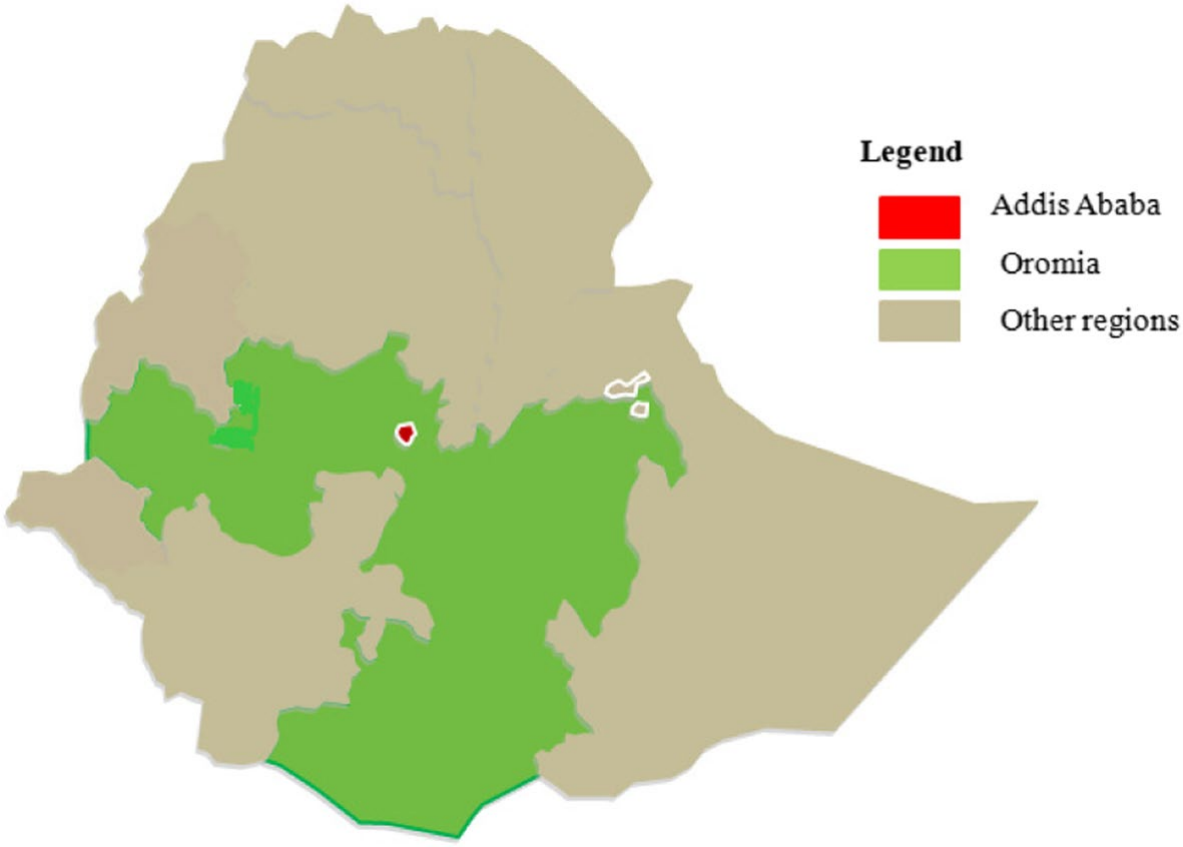
The map of Ethiopia showing the location of Addis Ababa, Oromia, and other regions

Addis Ababa (also called Finfinne among the Oromo) is the seat of the federal government of Ethiopia, the seat of African Union head quarter, and is also the capital of the Oromia regional state. The city is demographically composed of a multicultural and multilingual societies. The top three languages in the city are Amharic (70.9%), Afaan Oromoo (10.7%) and Gurage (8.3%), according to the 2007 census report [22]. Although geographically located in Oromia regional state, surrounded by the Afaan Oromoo-speaking communities in all directions (see Fig. 1) and also having many Afaan Oromoo-speaking residents, the city uses only Amharic as an official language for government services including healthcare delivery [21]. Moreover, the demographic makeup of Addis Ababa is rapidly changing particularly due to internal migration [23]. However, there is limited evidence showing health policy efforts addressing the health needs of the city’s growing non-Amharic speaking population.

Even though there is strong evidence from different parts of the world concerning the impacts of language barriers on access, quality, and safety of healthcare [2, 3, 24, 25], little research has been conducted on the experience of those refused receiving healthcare in their language in Ethiopia. We identified only one published study assessing the impacts of language barriers in accessing healthcare services for Afaan Oromoo-speaking population in Ethiopia [19] and no published work has thus far thoroughly examined this topic in Addis Ababa. However, there are some evidences pointing out the existence of effects from language barriers in general on the healthcare access in Addis Ababa [20, 26, 27]. Focusing on Addis Ababa is needed for many reasons, among which the main one are: the city is the capital of Oromia although it does not use Afaan Oromoo as a working language, the hospitals in the city are meant to serve both Afaan Oromoo-speaking resident population and large number of those coming from the surrounding areas in Oromia through referral system, and finally, many specialized healthcare services in the country are provided only in hospitals found in the city. Therefore, the general objective of this study is to explore the impacts of language barriers on access and quality of healthcare services received among Afaan Oromoo-speaking patients in Addis Ababa, Ethiopia.

## Methodology

### Study design

Institution-based, exploratory study using a qualitative approach was carried out from August 2021 to January 2022 to examine the impacts of language barriers on the access and quality of healthcare services for Afaan Oromoo-speaking patients in 6 public hospitals in Addis Ababa.

### Sampling and participants

The participants of the study were Afaan Oromoo-speaking patients who reported to have barriers to communicate in Amharic. The inclusion criteria for the patient participants were being Afaan Oromoo speaker, having self-reported barrier to speak in Amharic, aged 18 years old or above (in the case of underage patients, using their attendants who are 18 years old or above) and having willingness to participate in the study. Patients fulfilling the criteria but whose medical and psychological conditions do not allow them to participate in the study were excluded. To recruit the patients, purposive sampling was used, and healthcare providers were involved in identifying and linking to data collectors the patients fulfilling the criteria listed above. While recruiting the participants, the principle of maximum variation sampling was employed to make sure that the experience of patients with different sociodemographic backgrounds (sex, age group, religion, and education) are included. The study also included healthcare providers to examine their experiences and perspectives concerning language barrier in their healthcare provision. Purposive sampling was employed to recruit experienced healthcare providers (those served in the hospitals in Addis Ababa at least for 3 years), and those speaking only Amharic and both languages (Afaan Oromoo and Amharic). The inclusion of both patients and healthcare providers were meant to triangulate and validate the reported language barriers related problems.

This study implemented the principle of information power to decide the number of interview participants [28]. Generally, 27 patients and 9 key informants comprising physicians participated in the study. From the key informants, 4 were Amharic speakers and 5 were both Afaan Oromoo and Amharic speakers. The socio-demographic characteristics of the patients or their attendants participated in the in-depth interviews are presented in Table 1. The term attendant refers to the parents or guardians having the language barrier problem and accompanying patients under the age of 18.

**Table 1:** Socio-demographic characteristics of the in-depth interview participants

***Socio-demographic variables***	***Frequency***	***Percentage***
***Sex***		
Male	17	63.0
Female	10	37.0
***Age group***		
18–25	4	14.8
25–34	6	22.2
35–44	11	40.7
45–54	4	14.8
> 54	2	7.4
***Religion***		
Orthodox Christian	15	55.5
Muslim	5	18.5
Protestant Christian	7	26.0
***Education***		
Non-literate	3	11.1
Elementary school	12	44.4
High school	7	26.0
Above high school	5	18.5

### Data collection procedure

A semi-structured interview guide was used to generate data about the study participants’ experience of language barriers, and effects on healthcare quality and access (see supplementary 1). The interviews were in-depth, face-to-face, conducted individually, and took place at the mutually agreed up on locations in the premises of the selected hospitals. The locations of the interviews were decided considering the need to keep the privacy of the participants information. Each interview lasted about 40 minutes to 1 hour and was audio-recorded after getting the consent of each study participant. The interviews with the patients or their attendants were conducted in Afaan Oromoo and for the healthcare providers, either Afaan Oromoo or Amharic was used depending on their language preference. The interviewers were ABO^2^ and DHR, both are healthcare professionals by training, and they are fluent speakers of both Afaan Oromoo and Amharic languages.

### Data analysis

The recordings of the interviews were transcribed verbatim and then translated into English for analysis by the authors. The process of translation involved attention to both finding equivalent terms and phrases in English with the Afaan Oromoo and Amharic versions of the interviews and understanding the context of the interviews. The transcripts of the interviews were checked for correctness and completeness through comparing the transcripts with the audio-recorded voices. Each transcript was read carefully to grasp the whole experience of the study participant regarding the topic under the study. After the whole transcript were read, line-by-line coding was conducted. Codes were then sorted into categories on the bases of their similarities, themes were then developed from the categories and finally thematic analysis technique was employed. Quotes that best describe the various themes were selected and presented in the results section. While presenting verbatim quotes from the interviews, KII and IDI abbreviations are employed in the results section to refer to Key Informant Interviews and In-Depth Interviews, respectively. To anonymize the hospitals participated in the study, abbreviations H1, H2… are employed in the data analysis section to refer to Hospital 1, Hospital 2, and so on.

### Ethical considerations

Written informed consent was received from each of the study participant for conducting the interviews after explaining purpose of the study and the study methods. All the personal profile of the study participants remained confidential, and anonymity of the findings are kept. The process of keeping confidentiality involved recording the personal profiles of patients only on papers and audio-recording only their answers pertaining to language barrier issues. In addition, the audio-records of the interviews are stored in a de-identified manner and are password protected. Ethical clearance for conducting the study was received from the College of Social Sciences and Humanities Research and Post-graduate Coordinating Office of Jimma University. The methodology employed in this study followed the principles of the Helsinki Declaration.

## Results

### Existence of language barriers

The existence of language barriers in the public hospitals in Addis Ababa has been stated both by the patients and healthcare providers. The healthcare providers stated that majority of the patients who have a barrier to communicate in Amharic speak Afaan Oromoo as their mother tongue. A physician at H1 (KII 4, Afaan Oromoo-speaking) estimated that more than 50% of the patients receiving medical care at the hospital are Afaan Oromoo speakers. Many of these patients come from the nearby places in Oromia region through referral system and most of them speak only Afaan Oromoo. They face problems emanating from language barriers all along the way starting from the entrance gate of the hospitals, and the healthcare providers explained that the issue of language barriers has been limiting the ability to take good quality patient history.

Interviews made with the patients and their attendants also show the existence of language barriers. For instance, IDI 8 (a woman) stated:

I cannot speak Amharic. I cannot understand what the physicians ask me, or I cannot tell them the health problem of my child. My interaction with the physician is moderated by the patients or other attendants who can speak both Afaan Oromoo and Amharic.

Similarly, IDI 12 (a man) stated:

When I go to pharmacy, I cannot communicate with the pharmacists. I have to wait until somebody who speaks both Afaan Oromoo and Amharic comes…Can a human and a tree communicate? It is like that. I cannot understand them; they cannot understand me.

Key informants stated that about 80% of the health assessment they make depends on the explanation the patients make about their health problems. If there is a language barrier, more reliance on lab tests has been reported to diagnose patients although that allows to know about the patient only 20%.

In all the hospitals where the interviews were conducted, ad hoc interpreters were used to ease language barriers. Ad hoc interpreters are searched from the patients, attendants, passing by people or healthcare providers who can speak both Amharic and Afaan Oromoo. It is stated that if these ad hoc interpreters are not available, the healthcare providers face huge obstruction in properly diagnosing and treating patients. The healthcare providers stated their worry in that the ad hoc interpreters may not ask questions the way the physician is asking, and they may also not explain the feeling of the patients adequately. There are many times when the healthcare providers or the patients are not able to find even the ad hoc interpreters. In such circumstance, a body language is used for making communication between the patients having language barriers and the physician. The case of a patient, IDI 18 (a man), is presented as follows,

I do have experiences of getting back to my home without getting the treatment I needed because of language barriers and due to not getting an ad hoc interpreter. To overcome language barriers, I sometimes rely on body language. Because I didn’t learn sign language and the physicians also have that problem, proper communication cannot take place this way. Our fathers say *yoo dhukkuba ofii himatan qoricha argatu* [it is only when you tell your disease that you get cure].

Patients or their attendants also described that they try their level best to explain what they feel and what they want to communicate using their very limited knowledge of Amharic.

### The impacts of language barriers on the provision of health services

It was found that language barriers result in many problems for both the patients and healthcare providers. These problems are presented below under the themes: preventable medical errors, adherence to treatment and health seeking behavior, additional cost to the patients, increased length of hospital stays, weak patient-provider therapeutic relationship, social desirability bias, ethical dilemma, patients are ashamed and feel less confident, dissatisfaction and anxiety, and added burden on the healthcare providers.

### Preventable medical errors

Regarding preventable medical errors, the following quote from KII 5 (physician, Amharic-speaking) indicates the case of a girl who underwent a surgery:

…The case was life threatening. I had a patient who underwent a surgery to remove an anomaly on her lung. We then performed chest tube for her. To remove the chest tube, we gave them an appointment with the help of ad hoc interpreter, and they went home. We believed that they have understood everything we told them. But they came back after 3 months, long time after their appointment date with a very critical condition, and a surgery was performed for the second time. We were blamed for not properly informing the patient about the follow up. Had we professional interpreter, problems of this kind could not happen.

KII 1 (physician, Afaan Oromoo-speaking) was also asked the question of whether he has observed patients with language barriers and whether medication problem has happened because of that. His response worth lengthy quote and it is presented as follows:

You are asking me something like spooning only once from an ocean. On any given day, I just give support for many patients having this problem. I am sympathetic about all of them. So, it is so difficult to separate only the case of one patient and present it as an example.

KII 1 (physician, Afaan Oromoo-speaking) continued,

...But simply let me tell you one very recent case; 1 month ago, at _____hospital *[the name of the hospital is removed]*. She had esophageal cancer. She came from Borana [*a place in Oromia*], she cannot speak Amharic. Esophagectomy was performed for her, and she told me that she had complication some days after the surgery. When asked about the time complication started, she told me it had been 3 days. That complication should have been treated as soon as she felt it. She told me that she had reported the complication to the physician with the help of an ad hoc interpreter. But the interpreter didn’t properly interpret and didn’t make the physician understand what happened.

The key informants explained that language barriers affect diagnosis and history taking. This, in turn, may lead to wrong diagnosis and ineffective treatment.

### Impact on adherence to treatment, appointment for follow up and health seeking behavior

Key informants replied that many patients with the language barrier come back after some time with their health condition not becoming better. When asked why they didn’t take the prescribed drug, their response is usually that they didn’t properly hear what was said.

KII 6 (physician, Amharic-speaking) stated the impact language barriers have on adherence to treatment as follows:

You see the existence of this problem mostly at the outpatient department when patients come for follow-up treatment. How the patient has been taking drugs could be completely different from how it was prescribed by the physician. This could be especially related to the amount prescribed and the sequence with food that the drug is prescribed to be taken. The patient may report that he had been ordered by the physician to take the drug after 30 minutes while the correct prescription from the physician may say 30 minutes before having food. These problems happen regularly.

The possible complications and side effects of treatments may not be properly explained to the patient because of language barriers. Regarding the interviews with the patients, some patients responded that they hardly understand the date of appointment for follow up because of the language barriers. They also stated that they face problems frequently in understanding prescriptions about the number of times in a day in which a drug is taken and the precautions to be taken, for instance, concerning food and side effects. In this regard, IDI 13 (a man) explained his experience as follows:

The physician and the pharmacist told me the frequency and number of pills to be taken in a day in Amharic, but I didn’t understand. I made a guess and started taking one pill at a time, three times a day. Because I couldn’t see any good progress with my health condition, I went to a local health facility [*outside of Addis Ababa, in Oromia region*]. The healthcare providers told me that the way I was taking the drugs was wrong and it was supposed to be two pills at a time, three time a day. Recovery from the illness took longer time as a result.

Language barriers have also been stated by the patients as a factor that leads to the discontinuation of treatment. Patients and attendants stated that they have faced challenges in understating the prescription about how many drugs to take in a day, at what time and foods that must be taken or not to be taken. The patients stated that problems they face due to language barriers discourage them to seek healthcare when they need it. IDI 10 (a woman) narrated her deep frustration as follows,

I have previously faced several terrible incidents related with language barriers. I am still facing the same problem. I am deciding that I will never come back. My health problem could be highly contagious or whatsoever, but I prefer dying in my bed than coming back here. Due to the problem of language barriers and the associated maltreatment I faced, there were times I even thought of holding electric wire to commit suicide.

### Additional cost to the patients

This study identified that high cost of treatment is associated with language barriers. If patients cannot explain their situation, the physicians involved in this study admitted that they may order a long list of laboratory tests believing that they could find by the lab tests the problems the patients couldn’t explain because of language barriers. This is reported to result in unnecessarily high cost for the patients. Both the healthcare providers and the patients replied that there are many times when treatment is delayed, and the patients are exposed for additional costs as a result. Patients also reported that they sometimes seek treatment at private hospitals where they know there are healthcare providers who speak their language although the private facilities cost them more than the public hospitals.

### Increased length of hospital stays

Patients who have language barriers stay longer in the hospital than others because of language barriers. The patient with a language barrier could find it difficult to know what facility is located where, because of which they usually spend more time in the hospitals.

IDI 14 (a man) responded:

Premise of the hospital is very wide, and I don’t know where to go because posts are not written in my language.

KII 4 (physician, Afaan Oromoo-speaking) explained that patient history taking is very important to understand their health problem. If that fails because of language barriers, the patient may stay in the hospital for a longer time without receiving proper treatment. Similarly, KII 5 (physician, Amharic-speaking) said:

When told to take laboratory test order to a laboratory room, they sit keeping it. That delays treatment.

The following verbatim from a patient, IDI 15 (a man), can also indicate how language barrier can extend hospital stay or how it can delay treatment.

When I need language interpretation service, I ask for passing by people or people waiting to receive treatment if they can speak Afaan Oromoo. If there is none, I have to wait for until one comes. There are times when I wait for hours. Even when I get somebody who is bilingual, he may say he is in hurry or not interested in interpreting. Not only me. Many patients are having this problem. If I fail to get any, I have to wait until those queued up are away and those serving are free. I can then try to make them understand what I need using all means of communication because they have free time.

Interview with patient IDI 23 (a woman) also affirms the impact of language barriers on hospital stay:

Failure to understand the said procedure exposes to unnecessary delay. I sometimes spend hours wandering here and there without knowing and understanding what is said, where to go and the like. Because of language barriers there are procedures that would take four to 5 days but that could have been finished within a day had a language barrier was not there.

### Weak patient-provider therapeutic relationship

When a patient cannot explain his/her problem because of language barrier, and ad hoc interpreter is used, the direct connection between the patient and the physician could be loosened, and this may put pressure on the physician to ignore the patient. According to KII 7 (physician, Amharic-speaking),

A physician should have a friendly relationship with the patient to take best quality patient history. If both the patient and the physician cannot speak the same language or if effective communication is not made due to language barriers, this friendly relation could be affected, and best quality patient history could not be generated.

On the side of the patients, IDI 23 (a woman) stated.

When I face a language barrier, I suspect what the physician is going to do on me. I believe two individuals who can’t communicate don’t feel sympathetic about each other.

### Social desirability bias

According to the key informants, there could be a disease or health condition which a patient doesn’t want to be known to their family members, for instance, sexually transmitted infections. In this case, the patient may intentionally hide his/her disease and history when a family member is assigned as an ad hoc interpreter. When a family member is assigned as an ad hoc interpreter also, he/she may not properly tell the patient when the medical condition the physician told him/her is very serious; for instance, end stage cancer. Healthcare providers also stated that the interpreter may oversimplify the health situation of the patient, and this may make the patient to neglect the precautions ordered by the physician.

### Ethical dilemma

Language barriers have serious implications for the healthcare ethics. When ad hoc interpreters are used, for instance, there is a high probability of violating the patient’s privacy. According to KII 2 (physician, Amharic-speaking),

As a physician, we have the duty to protect the privacy of patients. As per the principle, we cannot even discuss the condition of a patient with the family members without his/her permission. But language barriers force us to violate this ethical principle when a family member is used as an interpreter. This is awful.

KII 6 (physician, Amharic-speaking) stated that ad hoc interpreters may translate communications to other languages in the way they understood, but which could be wrong. She asked,

How can I know whether that person is properly translating what I or the patient is saying?

Concerning medico-legal issue, KII 2 (physician, Amharic-speaking) stated:

If medical malpractice happens because of language barriers, how can I be responsible for that? I may have given the patient proper treatment. But if the translator tells wrongly to the patient, how can I be blamed? The ad hoc interpreter is just causal, and he could not be very serious about what I am communicating to the patient.

### Patients are ashamed and feel less confident

According to the healthcare providers, when the patients fail to properly communicate about their health needs, and when at the same time, they are aware that other patients are smoothly communicating, they shift the blame on themselves; they consider themselves as weak. It is because of this perceived self-limitation that makes the patients feel ashamed and develop anxiety to communicate with healthcare providers. Patients participated in the study reported that they feel anxious when seeking treatment from hospitals in Addis Ababa due to language barriers.

KII 4 (physician, Afaan Oromoo-speaking) explained how a language barrier is exposing patients to the feeling of embarrassment and low self-image in the following way:

As we are speaking now, there is a mother in this ward who admitted her son for treatment, accompanied by her nephew. Her son was seriously ill when admitted but the mother and her nephew were very shy even to speak to anybody or to ask for help as they had a language barrier. They speak only Afaan Oromoo. Later, they brought somebody who could interpret language for them and received treatment for the child.

The healthcare providers explained the strong power such feelings could have in discouraging the healthcare seeking behavior of patients. Feeling less confident because of language barrier has the power to desist patients from expressing their pains and needs, even in emergency. IDI 2 (a woman), for instance said:

I was in labour but could not get registration card as soon as possible because I could not explain to the officers what I was feeling. Though seriously painful, I sat quietly because I feared they couldn’t understand my language.

### Dissatisfaction, anger and anxiety

Key informants explained that language barriers affect patient satisfaction. Interviews with the patients also show that they have been angered and felt desperate. The case of IDI 5 (a man) is presented as follows:

I am sitting here annoyed. The doctor asked me to bring my blood test result. The lab room workers took my card and ordered me to call the doctor who ordered the lab test, I think. I went back to the doctor, and the doctor asked me to give him my card which was already taken from me by those in the lab room. But I am observing those who speak Amharic have taken back their card from the lab room. I am in this deal for four hours. I am feeling desperate.

While explaining her similar experience in the hospital, another patient (IDI 16, a woman) said:

From their face [the physician] I understand that they have anxiety in speaking to patients who have language barriers. The same is true on the side of the patients. Patients having language barriers develop anxiety to speak to the physician. Sometimes, I see physician speaking loudly to me and I feel that they could be scolding.

While explaining dissatisfaction with the healthcare due to language barriers, a patient (IDI 23, a woman) stated that such negative experience has the capacity to discourage health seeking behavior as follows:

With my previous experiences of treatment in the hospitals in Addis Ababa, if I say I was satisfied, that is quite far from the truth. Because I didn’t find physicians who are able to understand what I talk to them, I don’t feel I have got adequate health service. I could get satisfying treatment if the government had assigned language interpreters. Otherwise, I prefer not coming back to these hospitals to receive the same healthcare that I was not satisfied with. I prefer dying in my bed at home than coming back.

The patients directed their dissatisfaction and anger not only at the healthcare facilities and healthcare providers. Some patients blamed the government for failing to provide healthcare in the language they understand. IDI 24 (a man) replied:

When you do not get services in your language in your own country where you also pay taxes, you feel very bad. This reduces your trust in your government and your bond to your country. That is because you don’t see practically the concept of equality you have been told in the constitution. This is not politics. I am talking facts.

### Added burden on healthcare providers

As stated above, one group of people who serve as ad hoc interpreters are healthcare providers. The key informants indicated that this voluntary service adds burden on them. Afaan Oromoo-speaking healthcare providers could be called while they are on other duties to provide language interpretation service when language barriers happen although there is no official recognition for such services. KII 8 (physician, Afaan Oromoo-speaking) elucidated the magnitude of the burden added on him because of language barriers as follows:

There are times when the Afaan Oromoo speaking healthcare providers are always called up to give language interpretation service for the patients who are not able to speak Amharic. There are times when this service appears our regular job because many patients who speak only Afaan Oromoo come here in large number. Many of our co-workers cannot speak Afaan Oromoo. Because of the friendship we have, those physicians seek interpretation assistance from Afaan Oromoo-speaking friends; to the extent of making a phone call and asking for interpretation support when we are away.

When asked whether any attempt was made to solve problems related with language barrier, KII 2 (physician, Amharic-speaking) stated:

Problems like this one are considered luxury as compared to the many other problems our hospital has. It is because of this interview that I even became conscious about the problem. Of course, I know that this is a part of human right. But we didn’t ever discuss it as a problem.

Another key informant, KII 9 (physician, Afaan Oromoo-speaking), stated that lodging official request to solve such problems needs knowing the bureaucratic procedure. As health professional, he responded:

We have rare contact with the government officials. We have frequent and direct encounter with the patients having language barriers, but we have been unable to present the existence of these problems and the need for solution to the concerned authorities.

KII 4 (physician, Afaan Oromoo-speaking) also explained his feeling about intervention as follows:

No intervention has been made. I didn’t also see while it was officially discussed. During the last 2 years, extensive catchment area has been added to this hospital from Oromia. But along with that, no consideration was given to the issue of language.

### Beliefs of patients about right to healthcare in one’s own language

Some patients consider their language barrier as a burden to the healthcare providers, and they consider it as a favor made to them when ad hoc interpreter is availed. On the other hand, many patients and their attendants indicated that most of them have the conviction that it is their right to access healthcare in their own language. A patient, IDI 15 (a man) stated,

It is very clear that everybody has the right to get healthcare in his own language. I believe that it is not my duty to learn the official language. It is the responsibility of the hospitals to provide service in the language that I understand. I pay for their service. Part of the oath that the physicians make is to serve all seeking treatment with equality, and it is their duty to keep that responsibility. As far as it is said nations and nationalities have the right to be served in their language, it is the duty of the government to provide the needed services in all the languages.

## Discussion

The study focused on the impacts language barriers bear on the provision of healthcare services for the Afaan Oromoo-speaking patients in hospitals found in Addis Ababa (Finfinne), Ethiopia. Although the qualitative nature of the study does not allow quantification, key informants in this study supported previous studies [20, 29] in stating that the majority of patients seeking health services in many of the public hospitals in Addis Ababa speak Afaan Oromoo. A previous study in one of the hospitals in Addis Ababa, for instance, states that 56.1% of the patients at the hospital speak Afaan Oromoo and 55.4% of them were unable to speak Amharic [29]. On the other hand, only very few of the healthcare providers were able to speak Afaan Oromoo [20, 29].

Despite the facts stated above, the hospitals in Addis Ababa use only Amharic as their working language, concordance with the Addis Ababa city government revised charter proclamation [30] and article 5(2) of the Ethiopian federal constitution [31]. Professional interpretation service is not instituted in the hospitals, like several other African countries [32–35]. Rather, ad hoc interpreters sourced from patients’ attendants, other patients, Afaan Oromoo-speaking healthcare providers, or any casual volunteers are used as a language broker between the patients and the healthcare providers. The use of ad hoc interpreters in the healthcare setting, however, carries risks of inaccurate language interpretation, misdiagnosis and wrong treatment [36, 37]. When nobody is around to interpret the communication between the patients and healthcare providers, it is reported that patients use body language to explain oneself.

The official recognition of only Amharic as a working language of the hospitals in the city is ironic given that the city is also the capital city of Oromia. The adverse impacts language barrier is having on the access to, and quality of healthcare constitutes structural violence. Structural violence refers to avoidable harms on individuals or communities because of the operations of social structures or institutions, and the consequences include failing to meet basic needs and unequal life chances [38]. In addition, the fact that Afaan Oromoo-speaking patients are denied access to healthcare in their language, in their own capital city, and in the hospitals they constitute significant portion of the patients implies serious breaching of moral and ethical standards, although this breaching was not emphasized in the health ethics studies and discussions in Ethiopia [39–42].

This study has identified many problems that are resulting from the language barriers in the hospitals of Addis Ababa. The major problems for the patients are: preventable medical errors, low treatment adherence, low health seeking behavior, additional treatment cost, increased length of hospital stays, weak therapeutic relation, social desirability bias, less confidence, and dissatisfaction with the healthcare. Results from studies conducted in different countries concerning the impacts of language barriers in healthcare settings confirms these findings [3, 25, 33, 35, 43]. The negative impacts of language barriers also extend to healthcare providers, as it is reported that burden is added to Afaan Oromoo-speaking healthcare providers playing the role of interpreters without any payment or official recognition for the service. Other studies also state that language barriers contribute to increased workload and workplace stress for the health professionals providing ad hoc interpretation services [34, 44]. Ethical dilemma also occurs in the situations of language barriers since ad hoc interpreters can access patients’ health information and due to the question of who takes responsibility for medication problems arising from wrong ad hoc interpretation.

Despite all the problems emanating from language barriers this study identified, the issue has not received adequate attention it deserves at both policy and practice level. This is in contrast with the article 49 (5) of the Ethiopian constitution which partly states that the state of Oromia has special interest in Addis Ababa regarding the provision of social services, which arguably could include health services in one’s own language [31] and WHO constitution stating that “The enjoyment of the highest attainable standard of health is one of the fundamental rights of every human being without distinction of race, religion, political belief, economic or social condition” [45]. It is very important to note here that there is absence of specific federal regulation which clearly indicate the right to access healthcare in one’s own language in Ethiopia, unlike the experiences of some multilingual African countries [46, 47].

As a solution to the impacts of language barriers in the healthcare settings in Addis Ababa, the study participants suggested the assignment of trained language interpreters who bridge the communication problems between the patient and healthcare provider. Moreover, giving priority in hiring multi-lingual healthcare providers and a political will on the side of the federal government to make Afaan Oromoo an additional working language of the hospitals are very crucial considering the significant number of patients speaking Afaan Oromoo, the geographical location of Addis Ababa being in Oromia, and constitutionally supported ‘special interest’ demand of Oromia in Addis Ababa in the provision of social services. As the last expedient, the authors suggest the use of translation technologies to address the problem with maximum care since such technologies carry more risks of wrong translation than professional human translators [48–50].

### Limitation of the study and implication for further studies

Since the study relied on a small sample size, it is impossible to grasp the exact magnitude of the impacts of language barriers on the healthcare access and quality for the Afaan Oromoo speaking patients in Addis Ababa. So, cross-sectional studies to assess the magnitude of the problem and advanced epidemiological studies to assess cause-effect are suggested. Further studies are also needed among patients with mental health problems since the diagnosis and treatment of mental health problems more relies on linguistic communication than on objective test and medication [51, 52]. Moreover, since health warnings for several products including tobacco and alcohol, and instructions for condom use are provided only in Amharic and English in Ethiopia, the impacts these might be having on those who cannot speak Amharic or English needs attention in future studies.

## Conclusion

The study reveals that many Afaan Oromoo-speaking patients seeking treatment in the public hospitals found in Addis Ababa are facing language barriers in accessing quality healthcare. The healthcare providers also stated that they have been challenged to take patient history and to communicate with the patients about the course of treatment. Despite the widely occurring problem of language barriers, officially designated ways of dealing with the challenge have not been implemented. Rather, methods like the use of ad hoc interpreters and body language are employed. Language barriers are found to have severe consequences on the quality of care, healthcare access, hospital stays, patients’ expenditure, and satisfaction level. The study recommends that the issue of language barriers should receive urgent attention among healthcare policy makers and healthcare administrators at both the federal and the city levels to enhance healthcare access and effectiveness, and to increase patient satisfaction. A political will is needed from the federal government and the city of Addis Ababa to make Afaan Oromoo an additional working language of the hospitals in Addis Ababa to address the language barriers problem affecting the Afaan Oromoo-speaking patients. In addition, the assignment of professional language interpreters, and mechanisms of hiring qualified multi-lingual healthcare providers who can serve patients with diverse linguistic background need to be established.

## Supplementary Information

**Additional file 1.** Interview guide.

## Data Availability

The anonymized interview transcripts used for the study are available upon reasonable request made to the corresponding author.
