# Supplementary material for: Impacts of language barriers on healthcare access and quality among Afaan Oromoo-speaking patients in Addis Ababa, Ethiopia
Source: BMC Health Serv Res. 2023 Jan 16;23:39. doi: 10.1186/s12913-023-09036-z (PMC9843916; doi:10.1186/s12913-023-09036-z)
Supplement: Supplementary file 1 — Additional file 1. Interview guide. [file 12913_2023_9036_MOESM1_ESM.docx]

**Interview guide for patients or their attendants**

**Socio-demographic information**

1. Sex of the participant_______________
2. Age ____________
3. Religion____________
4. Education___________
5. Name of the hospital_________

**Questions related to language barrier in the healthcare setting**

1. Do you have challenges in speaking Amharic?
2. Have you faced problems in trying to interact with healthcare providers because of language barrier?
3. Would you tell us specifically what problems you faced because of language barrier? For each identified problem, please ask how they overcame the challenge.
4. How do healthcare providers respond when they notice that you cannot speak their language?
5. Can you tell us the difference in the level of your satisfaction between when you encounter Afaan Oromoo speaking healthcare provider and non-Afaan Oromoo speaking healthcare provider?
6. Where is the place/service in healthcare facility where you find it most challenging to communicate because of language barrier?
7. Do you believe it is your right to receive healthcare services in your language?
8. What do you suggest to overcoming language barrier in healthcare facilities?

**Interview guide for the healthcare providers**

1. Sex __________
2. Years of serving in Addis Ababa__________
3. Language spoken__________
4. Name of the hospital ___________
5. Do you encounter problems related to language barrier in the hospital?
6. Would you tell us if Afaan Oromoo speaking patients who have difficulty speaking Amharic come to the hospital for healthcare? Probe for their number and where they come from.
7. Would you tell us the problems Afaan Oromoo speaking patients face due to language barrier? Probe for typical examples/ encounters they can tell.
8. Would you tell us the challenges such language barrier has for the healthcare providers? Probe for how they usually overcome the challenges.
9. Do you believe it is the right of the patients to be served in their language?
10. Would you tell us if any intervention has been made so far or if there is a regulation to address the challenges related to language barrier?
11. What do you suggest to overcoming the problems related to language barrier discussed in this interview?
